# An Unusual Case of Persistent Hypoglycemia in Scrub Typhus

**DOI:** 10.7759/cureus.21098

**Published:** 2022-01-11

**Authors:** Poonam Arora, Hari Prasad, Nishant Ranjan, Aadya Pillai, Naveen Joseph

**Affiliations:** 1 Emergency Medicine, All India Institute of Medical Sciences, Rishikesh, Rishikesh, IND

**Keywords:** scrub typhus, orientia tsutsugamushi, meningoencephalitis, hypoglycemia, blood sugar

## Abstract

Acute fever is a common worldwide issue among patients presenting to the emergency department. Main tropical fevers presenting as acute fever are malaria, dengue fever, typhoid, scrub typhus, and leptospirosis. Scrub typhus is a zoonotic disease caused by *Orientia tsutsugamushi*, which is endemic in many parts of India. Its clinical presentation varies significantly from undifferentiated fever and fever with thrombocytopenia to meningoencephalitis. Persistent hypoglycemia in scrub typhus has not yet been reported. Here we report a case of a 27-year-old male who presented with altered mental status after seven days of scrub typhus diagnosis. Diagnosis of hypoglycemia (random blood sugar of 37 mg/dL) was made at triage. Altered mental status responded to dextrose, but despite meals and treatment, hypoglycemia persisted. The cause for persistent hypoglycemia remained unexplained as most laboratory panels for hypoglycemia were either normal or did not fit into a particular cause.

## Introduction

Scrub typhus, caused by *Orientia tsutsugamushi*, is a common cause of acute fever in tropical countries. Scrub typhus has been reported in 23 out of 29 states in India [1]. Outbreaks are most commonly seen during monsoon and post-monsoon seasons (July to November) [2]. It usually presents as fever, myalgia, rash, jaundice, thrombocytopenia, hepatomegaly, and splenomegaly. The main complications are acute respiratory distress syndrome, hepatitis, acute kidney injury, myocarditis, and meningoencephalitis [3]. Here, we present a case of a 27-year-old male diagnosed with scrub typhus presenting with altered mental status and hypoglycemia. The patient's mental status improved, but hypoglycemia persisted despite the use of dextrose and steroids.

## Case presentation

A 27-year-old male without known comorbidities and not on any medications was brought to the emergency department with a complaint of altered mental status. Family members provided the history of high-grade fever with chills and rigor for one week. He also complained of right upper abdominal pain and non-bloody non-bilious vomiting for the past two days. There was no history of cough or shortness of breath. There was no history of travel to forests or places endemic to scrub typhus. Scrub typhus immunoglobulin (Ig)M assessed outside of our department was positive.

On examination, the patient was alert with the Glasgow Coma Scale of E2V3M5. His vital signs were: heart rate of 86 beats per minute, oxygen saturation of 97% in room air, blood pressure of 124/72 mm Hg, respiratory rate of 20 breaths per minute, and point of care blood sugar of 37 mg/dL. The abdomen was soft, distended, and non-tender on examination. All other findings were normal.

Initial laboratory investigations revealed thrombocytopenia, mild transaminitis, and raised urea and creatinine, as shown in Table 1. Ultrasonography of the abdomen revealed hepatomegaly with grade two fatty liver. Other radiological investigations were within normal limits.

**Table 1:** Initial laboratory parameters

Parameters	Results	Reference range
Hemoglobin	12.7 g/dL (7.88 mmol/L)	12–15 g/dL (7.45–9.31 mmol/L)
Red Blood Cell Count (RBC)	4.01 × 10^6^/mcL (4.01 × 10^12^/L)	3.8–5.2 × 10^6^/mcL (3.8–5.2 × 10^12^/L)
White Blood Cell Count (WBC)	8.7 × 10^3^/mcL (8.7 × 10^9^/L)	4–11 × 10^3^/mcL (4–11 × 10^9^/L)
Platelets	34 × 10^3^/mcL (34 × 10^9^/L)	150–400 × 10^3^/mcL (150–400 × 10^9^/L)
Total bilirubin	2.2 mg/dL (37.62 µmol/L)	0.3–1.2 mg/dL (5.13–20.52 µmol/L)
Direct bilirubin	0.8 mg/dL (37.62 µmol/L)	0–0.2 mg/dL (0–3.42 µmol/L)
Alanine aminotransferase (ALT)	88 U/L (1.46 µkat/L)	0–35 U/L (0–0.58 µkat/L)
Aspartate aminotransferase (AST)	109 U/L (1.81 µkat/L)	0–35 U/L (0–0.58 µkat/L)
Alkaline phosphatase (ALP)	742 U/L (12.32 µkat/L)	30–120 U/L (0.5–2 µkat/L)
Serum Albumin	3.5 g/dL (35 g/L)	3.5–5.2 g/dL (35–52 g/L)
Urea	116 mg/dL (41.41 mmol/L)	17–43 mg/dL (2.83–7.15 mmol/L)
Creatinine	2.56 mg/dL (226.36 µmol/L)	0.55–1.02mg/dL (48.63–90.19 µmol/L)
Sodium	135 mEq/L (135 mmol/L)	136–146 mEq/L (136–146 mmol/L)
Potassium	4.1 mEq/L (4.1 mmol/L)	3.5–5.1 mEq/L (3.5–5.1 mmol/L)

Therefore, the patient was started on doxycycline (100 mg twice daily) intravenously and supportive treatment. During the hospital stay, blood sugar was charted every two hours. The patient's random blood sugar values were persistently between 70-100 mg/dL with some reading below 70 mg/dL, but the patient did not report any symptoms. Hypoglycemia persisted despite dextrose and hydrocortisone administration at a level of no more than 110 mg/dL as random blood sugar monitoring, as shown in Table 2. Thus, a hypoglycemia investigations panel was sent. A 72-hour fasting test was done, followed by sending serum insulin and C peptide. Hypoglycemia symptoms, such as palpitations, sweating, and confusion, among others, were explained to the patient as well as his attendants. He was also asked to report any symptoms during the fasting period. Critical samples were drawn when glucose was less than 60 mg/dL, immediately followed by 100 ml of 25% dextrose infusion. The patient did not complain of any symptoms, even when blood sugar was 58 mg/dL. Reports of hypoglycemia panel investigations are shown in Table 3.

**Table 2:** Blood sugar monitoring in mg/dL during admission

Time	Day 1	Day 2	Day 3	Day 4	Day 5	Day 6	Day 7
6 am		70	96	72	100	75	70
8 am		90	70	80	92	58	80
10 am		56	101	88	67	80	56
12 pm		100	104	76	82	83	70
2 pm		74	76	62	84	86	92
4 pm		32	68	92	70	51	98
6 pm		98	79	94	65	80	
8 pm		76	68	70	88	73	
10 pm		70	94	64	82	81	
12 am	37	41	66	88	63	91	
2 am	24	107	80	100	79	64	
4 am	87	101	96	68	92	74	

**Table 3:** Hypoglycemia panel of investigations T3, triiodothyronine; T4, thyroxine

Test	Results	Reference range
Fasting plasma glucose	58 mg/dL	70–110 mg/dL
Free T3	4.86 pg/mL	2–4.4 pg/mL
Free T4	1.47 ng/dL	0.85–1.86 ng/dL
Growth hormone	2.45 ng/mL	≤ 10 ng/mL
Cortisol	9.20 µg/dL	5–25 µg/dL
Insulin	2.39 mU/L	2.6–37.6 mU/L
C peptide	0.86 ng/mL	1.10–4.4 ng/mL
Insulin-C peptide molar ratio	0.0604	1

The insulin-C peptide ratio was 0.0604, showing hypoinsulinemic hypoglycemia rather than hyperinsulinemic hypoglycemia seen in insulinoma and insulin autoimmune syndrome (Hirata's disease). Insulin-like growth factor and antibodies against it were unavailable for testing; hence, no data. The patient improved symptomatically; thus, he requested a discharge. Warning signs and home treatment of hypoglycemia were explained to him, and he was discharged. Random blood sugar at discharge was 98 mg/dL with no symptoms of hypoglycemia. The patient was called for follow-up after seven days, and random blood sugar was 94 mg/dL without any symptoms in the last seven days.

## Discussion

Scrub typhus was first reported in Kumaon hills (Uttarakhand) in 1938 [4]. Scrub typhus is an acute febrile illness caused by *Orientia tsutsugamushi* with frequent multiorgan involvement. It is transmitted by the bite of infected chiggers of thrombiculid mite. The main targets of *O. tsutsugamushi* are endothelial cells and monocytes, infection of which leads to endothelial dysfunction and vasculitis [5].

Common clinical symptoms include fever with chills, shortness of breath, jaundice, altered mental status, vomiting, abdominal pain, cough, rash, and myalgia. Other rare features include melena, convulsions, and generalized body edema. Common laboratory abnormalities are transaminitis, thrombocytopenia, hypoalbuminemia, hyponatremia, anemia, hypokalemia, hyperkalemia, leucopenia, and less commonly, hypernatremia [6-8]. Hypoglycemia has not yet been reported in the literature as a presentation or laboratory abnormality in scrub typhus. Our patient had hypoglycemia resistant to dextrose or steroids.

In a patient presenting with hypoglycemia, the first step involves confirming the diagnosis by Whipple's triad: symptoms or signs of hypoglycemia, low plasma glucose concentration (≤ 70 mg/dL), and resolution of those symptoms or signs after the plasma glucose is raised by glucose administration [9]. The second step is to assess serum insulin and C peptide concentrations during a hypoglycemic episode, i.e., a laboratory blood glucose value of less than 50 mg/dL [10]. Other investigations required are serum cortisol, growth hormone, and thyroid function tests. Hypoglycemia is then classified as hypoinsulinemic hypoglycemia (hormone deficiency of cortisol, growth hormone, or thyroid hormones; insulin receptor antibodies, abnormalities of insulin-like growth factor, liver disease, etc.) and hyperinsulinemic hypoglycemia (exogenous insulin, oral hypoglycemic agents, Hirata's disease, nesidioblastosis, and insulinoma) [11-13].

Hypoglycemia treatment involves administering intravenous dextrose 1 g/kg in adults and 0.5-1 g/kg in children (50% dextrose in adults, 25% dextrose in children, and 10% dextrose in neonates) [14]. Other treatment modalities include administration of corticosteroids, somatostatin analogs like octreotide, and diazoxide and even pancreatectomy [15,16].

## Conclusions

Scrub typhus has varied clinical presentations from undifferentiated fever, bilateral pneumonia resembling atypical pneumonia, or fever with diverse sepsis syndromes. Persistent hypoglycemia is not yet reported in a case of scrub typhus. Knowledge of this will help rule out infectious causes as a cause of recurrent unexplained hypoglycemia. No patient should leave the emergency without proper evaluation of the etiology of hypoglycemia and the problem should be addressed at each level, as hypoglycemia is associated with considerable adverse outcomes in many acute critical illnesses. A timely health education program and close monitoring should be done to reduce hypoglycemia-associated morbidity and mortality.
